# Vitamin D as a Resilience Factor, Helpful for Survival of Potentially Fatal Conditions: A Hypothesis Emerging from Recent Findings of the ESTHER Cohort Study and the CHANCES Consortium

**DOI:** 10.3390/nu7053264

**Published:** 2015-05-06

**Authors:** Ben Schöttker, Hermann Brenner

**Affiliations:** German Cancer Research Center (DKFZ)-Division of Clinical Epidemiology and Aging Research, Im Neuenheimer Feld 581, 69120 Heidelberg, Germany; E-Mail: h.brenner@dkfz.de

**Keywords:** vitamin D, mortality, death, cardiovascular disease, cancer, review

## Abstract

There is debate on whether vitamin D deficiency is a risk factor for major chronic diseases and premature death or whether observed associations were just confounded by general health status. Here, we review recent results from the Epidemiologische Studie zu Chancen der Verhütung, Früherkennung und optimierten Therapie chronischer Erkrankungen in der älteren Bevölkerung (ESTHER) cohort study and the Consortium on Heatlh and Ageing: Network of Cohorts from Europe and the United States (CHANCES) that suggest that vitamin D deficiency may not be a risk factor for the development of cardiovascular diseases and cancer but may be a risk factor for fatal instances of these diseases. Furthermore, analyses comprehensively adjusted for the health status showed that the association of vitamin D and mortality was very likely not confounded by general health status. These results suggest that vitamin D could be a marker of resilience to fatality of potentially fatal diseases. Sufficient vitamin D serum concentrations may be needed to regulate the response of the immune system when it is challenged by severe diseases to prevent a fatal course of the disease. If this hypothesis can be verified through basic research studies and adequately designed randomized controlled trials, it could have important public health implications because vitamin D deficiency is very common worldwide, and interventions could be implemented easily.

## 1. Introduction

Although vitamin D is obtained from diet and dietary supplements, the main source for vitamin D is its production in the skin under the influence of solar ultraviolet B (UV-B) radiation. Therefore, the active vitamin D metabolite 1,25-dihydroxyvitamin D (1,25(OH)_2_D) should be considered rather as a hormone than a vitamin. The early chemical stages are produced in the skin [1], are metabolized to 25(OH)D in the liver [2] and are hydroxylated to the active form 1,25(OH)_2_D in the kidneys [3].

Although not the active metabolite, the most commonly measured vitamin D metabolite is serum 25-hydroxyvitamin D (25(OH)D) due to its higher half-life (~3 weeks) and up to 1000-fold higher serum levels compared with the physiologically active metabolite 1,25-dihydroxyvitamin D (half-life: a few hours) [4].

The active metabolite, 1,25(OH)_2_D binds to the vitamin D receptor, which is expressed in numerous organs such as the intestine, bones, kidneys and in many other tissues and cell types [5], including immune cells [6], cardiac myocytes [7] and cancer cells [8]. Therefore, 1,25(OH)_2_D could have a preventive effect on adverse conditions via binding at the vitamin D receptor and, vice versa, vitamin D deficiency and insufficiency may act as risk factors for several chronic diseases, including osteoporotic diseases [9,10], cardiovascular diseases (CVD) [11], diabetes mellitus [12], several types of cancer [13], infections [14] and several auto-immune conditions [15].

In high-income countries, CVD and cancer cause the majority of premature deaths [16]. If a role of vitamin D in the development of these two major diseases can be established, vitamin D would consequently be a risk factor for premature death. However, there is debate on whether vitamin D deficiency is a risk factor for CVD, cancer and premature death or whether associations observed in epidemiological studies were just confounded by the general health status because subjects with poor health have lower 25(OH)D levels. 

This review will focus on five recently published papers with results from the large Epidemiologische Studie zu Chancen der Verhütung, Früherkennung und optimierten Therapie chronischer Erkrankungen in der älteren Bevölkerung (ESTHER) study because this allows a direct comparison of results for different non-fatal and fatal outcomes, whereas reviews over several studies face problems of heterogeneity of study populations [17,18,19,20,21]. For analyses on mortality endpoints, ESTHER data were combined with data from seven other population-based cohort studies from the Consortium on Health and Ageing: Network of Cohorts in Europe and the United States (Consortium on Health and Ageing: Network of Cohorts in Europe and the United States (CHANCES); www.chancesfp7.eu; [21]) to validate earlier published findings of the ESTHER study alone [20].

In the following chapters, we very briefly summarize the results from the ESTHER cohort and the CHANCES consortium on the associations of 25(OH)D levels with CVD (fatal and non-fatal), cancer (fatal and non-fatal) and total mortality and explain why the hypothesis that vitamin D could be regarded as a resilience factor, helpful to survive potentially fatal conditions, crystallized as the overall interpretation of the findings. 

## 2. Methods

The ESTHER study is a population-based cohort study of older adults, which has been described in detail elsewhere [20]. In brief, 9949 participants were recruited in the German federal state of Saarland between July 2000 and December 2002 by their general practitioner (GP) during a regular health check-up. Follow-up questionnaires, including information on incident diseases (e.g., CVD and cancer), were sent to study participants and their GPs after 2, 5 and 8 years with response rates among survivors of 96%, 87% and 79%, respectively. Data on incident cancer cases were also available from the Saarland Cancer Registry and were used to validate and complete physician-reported cancer cases. 

To make CHANCES cohorts more comparable, analyses were restricted to study participants aged 50–79 years. The included cohorts with countries of origin and included sample size were: The ESTHER study (Germany, *n* = 9083), the National Health and Nutrition Examination Survey (NHANES) III (USA, *n* = 5626), the Tromsø study (Norway, *n* = 4406), the Health, Alcohol and Pychological factors in Eastern Europe (HAPIEE) studies from Czech Republic (*n* = 2029), Poland (*n* = 1700) and Lithuania (*n* = 1574), the Monitoring of Trends and Determinants in Cardiovascular Disease (MONICA)- Cooperative Health Research in the Region of Augsburg (KORA) study (Germany, *n* = 939) and the Survey in Europe on Nutrition and the Elderly; a Concerted Action (SENECA) (recruited in 12 European countries, *n* = 661). The cohorts’ key characteristics are described elsewhere [20,22,23,24,25,26]. Overall, 26,018 study participants with 25(OH)D measurements could be analyzed in the CHANCES analysis.

Serum 25(OH)D levels were measured with immunoassays (for details see reference [21]) in serum samples collected before disease onset and all analyses were true prospective analyses. In some analyses the definitions of the US-American Institute of Medicine (IOM) were used to define vitamin D deficiency (<30 nmol L^−1^ 25(OH)D) and vitamin D insufficiency (30–50 nmol L^−1^ 25(OH)D) [27] and in other analyses, quintiles or season of blood donation-standardized quartiles were used. Sensitivity analyses showed that results were always comparable for vitamin D deficiency and for the lowest quintile or quartile (irrespective if adjusted or standardized for season). Reported results from the ESTHER cohort and the CHANCES consortium for vitamin D deficiency and the lowest 25(OH)D quintile or quartile are therefore comparable. The reference group was always the highest 25(OH)D category, except for the analyses on cancer incidences that used the 25th to 75th percentile of 25(OH)D levels as the reference in order to look into potentially increased cancer risks in the bottom and top quartile of 25(OH)D levels. There was no increased cancer risk in the broad middle of the distribution of 25(OH)D levels in the ESTHER cohort which allowed the creation of this large reference group in order to increase the statistical power of the analysis. Furthermore, results for the bottom 25(OH)D quartile did not change in a way that it would lead to a different interpretation of the results if the highest 25(OH)D quartile was used as the reference. All analyses were adjusted for at least age, sex, season of blood draw, education, body-mass index (BMI), smoking and physical activity. If several different models were carried out, we only show the results from the most comprehensively adjusted model and the analysis with the longest follow-up time (*i.e.*, highest case numbers). A limitation of the analyses is that they were not adjusted for dietary factors but this applies for all end-points studied and cannot explain differences for the outcomes. For more detailed methods, we refer to the original articles that have been published elsewhere [17,18,19,20,21]. 

## 3. Is Vitamin D Deficiency a Risk Factor for CVD and Cancer?

**Figure 1:**
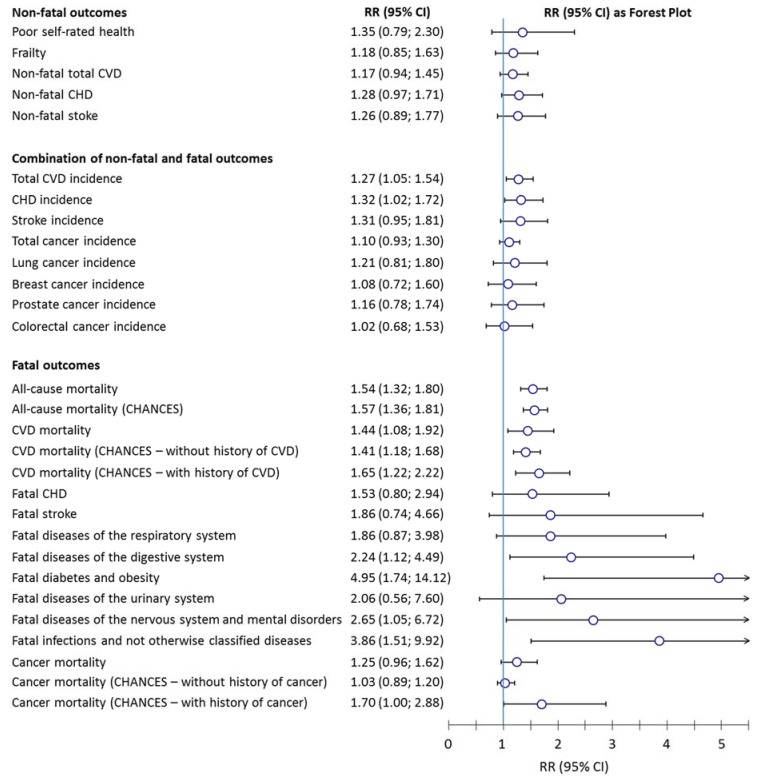
Association of lowest *vs.* highest 25-hydrodroxyvitamin D category^1^ with different prospective outcomes in the ESTHER study (if not stated otherwise). CHANCES, Consortium on Health and Ageing: Network of Cohorts in Europe and the United States; CHD, coronary heart disease; CI, confidence interval; CVD, cardiovascular disease; RR, risk ratio; ^1^ 25(OH)D < 30 nmol L^−1^ (*i.e.*, vitamin D deficiency) *vs.* 25(OH)D ≥ 50 nmol L^−1^ (*i.e.*, vitamin D sufficiency) or bottom quintile/quartile *vs.* top quintile/quartile of 25(OH)D levels. Exceptionally, in analyses on cancer incidences the 2nd and 3rd quartile combined were used as the reference group. However, sensitivity analyses showed that analyses with 25(OH)D quintiles or quartiles and clinical cut-offs yielded comparable results in the Epidemiologische Studie zu Chancen der Verhütung, Früherkennung und optimierten Therapie chronischer Erkrankungen in der älteren Bevölkerung (ESTHER) study. Note: Results from the most comprehensively adjusted model and with the longest follow-up time (*i.e.*, highest case numbers) are always shown.

Figure 1 shows that 25(OH)D levels were neither significantly associated with non-fatal total CVD, coronary heart disease (CHD), stroke nor with cancer incidence end-points in the total ESTHER study [17,18]. 

A significant association in the total cohort was only observed for fatal CVD (in ESTHER and CHANCES analyses) and cancer mortality in participants with a history of cancer (in CHANCES analysis) [17,20]. Furthermore, the association of low 25(OH)D with total CVD and CHD incidence was borderline significant, but this excess risk can be ascribed to the fatal events in these outcomes that combine non-fatal and fatal events. The fact that the associations of 25(OH)D with non-fatal cardiovascular outcomes were weak and not statistically significant in the same cohort indicates that vitamin D deficiency may not be a risk factor for the development of CVDs but may have an impact on their prognosis. The results for cancer point in the same direction: in the CHANCES analysis, 25(OH)D levels were only associated with cancer mortality in participants with a history of cancer at the time of recruitment but not in those without a history of cancer (Figure 1) [21]. In the ESTHER analysis with total cancer incidence and cancer site specific incidence endpoints, no associations were observed in the total cohort (Figure 1) [18]. This speaks for an important role of vitamin D in cancer prognosis rather than a causal relationship between vitamin D deficiency and early phases in the carcinogenic process. 

## 4. Is Vitamin D Deficiency a Risk Factor for Mortality?

The results from the CHANCES consortium including the ESTHER cohort showed that low 25(OH)D levels were consistently associated with all-cause mortality in cohorts from the USA, Northern, Eastern and Central Europe [21] and confirmed previous findings from the ESTHER study alone (Figure 1) [20]. With exception of cancer mortality that should be analysed stratified by history of cancer as shown in the CHANCES analysis and some causes of deaths with low statistical power, vitamin D deficiency was also significantly associated with many specific causes of mortality in the ESTHER study (Figure 1) [19]. Overall, the associations of low 25(OH)D levels with fatal outcomes were much stronger (risk ratio point estimates > 1.4) than with non-fatal outcomes or combinations of non-fatal and fatal outcomes (risk ratio point estimates ≤ 1.35), when analyses of cancer mortality that included participants without a history of cancer at time of recruitment were not regarded because they are a special case (Figure 1). It should be noted that analyses on cancer mortality restricted to study participants with a history of cancer are a special case as well and could be prone to reverse causality because the cancer could affect the 25(OH)D levels. It is a possible scenario that subjects with more hazardous cancer types or stages have lower 25(OH)D levels than subjects with less hazardous cancer types or stages because of the cancer and have a poorer prognosis because of the cancer type or stage rather than because of the low 25(OH)D levels. We did not adjust for cancer type and cancer stage because we did not have that information available in the CHANCES consortium and cannot exclude reverse causality. However, a recent systematic review of other cohort studies focussing on the prognostic value of 25(OH)D levels in well-characterized breast or colorectal cancer patients [28] supports our finding because the majority of the included studies and the meta-analyses of the study results also indicated a poorer cancer prognosis of cancer patients with low 25(OH)D levels.

Furthermore, the association of vitamin D with mortality could be confounded by the general health status because subjects with poor health have lower 25(OH)D levels and it is difficult to comprehensively adjust for the general health status in epidemiological studies. Therefore, in the ESTHER paper “Is vitamin D deficiency a cause of increased morbidity and mortality at older age or simply an indicator of poor health?”, we tried to disentangle the contributions of 25(OH)D and a poor health status to premature death [19]. Poor health was defined by two complementary markers: the subjective marker “Self-rated health (SRH)” and the objective marker “frailty”. Frailty is an emerging concept in aging research and reflects a multidimensional syndrome of loss of reserves (energy, physical, ability, cognition, and health) that gives rise to vulnerability [29]. In the cited analysis of the ESTHER study, frailty was assessed by a continuous frailty index at all ESTHER contacts (for a longitudinal analysis) according to the method of Mitnitski and Rockwood that defines frailty by an accumulation of deficits [30]. Frailty and SRH are known to be strongly associated with mortality [31,32].

Vitamin D deficiency and insufficiency were associated with SRH and frailty in cross-sectional but not in longitudinal analysis (Figure 1). Therefore, vitamin D deficiency could be regarded as an indicator of poor health but likely does not cause this condition. The other direction from frailty or a poor health state to vitamin D deficiency is the more plausible direction because subjects with poor health or frailty typically spend less time outdoors due to an overall lower rate of activity [33], limiting their production of provitamin D_3_ in the skin under solar ultraviolet B radiation, which is the main determinant of 25(OH)D levels [34]. Furthermore, senescent skin cells have a limited capacity to produce provitamin D_3_ [35]. It can be assumed that subjects with senescent skin cells have senescent cells also in other tissues and are at a higher risk of poor health and especially frailty. Both sun-avoiding behavior and senescent skin cells could explain lower 25(OH)D levels among subjects with poor health. This hypothesis was supported by the finding that higher age (maybe a proxy for senescent (skin) cells), lower physical activity and weight loss (both frailty components) were strongly associated with the strongest decrease in 25(OH)D levels during follow-up in the ESTHER study [19].

Nevertheless, 25(OH)D was associated with mortality outcomes in the ESTHER study, despite comprehensive adjustment for SRH and frailty [19], which was in line with two recently published studies, which also observed a persisting association in models adjusted for baseline frailty [36,37]. From the observation, that vitamin D deficiency was useful for further mortality risk stratification among subjects with poor health, the authors of this review were able to even confirm additive effects of low 25(OH)D levels and frailty on mortality [37].

Taken together, there are strong indications that low 25(OH)D levels do not cause a poor health status and seem to be in part a consequence of frailty. However, low 25(OH)D levels cannot be in total be explained by the health status because otherwise, vitamin D deficiency could not be associated with mortality independent from the general health status. 

If vitamin D deficiency does not lead to the precursor of death, a poor health status, this raises the question as to whether the observed association of vitamin D deficiency and mortality can be causal. Below, the pros and cons will be discussed with Hill’s criteria for a causal relationship: strength, consistency, biological gradient, specificity, temporality, plausibility, coherence, experiment and analogy [38].

### 4.1. Strength

The observed approximately 1.6-fold increased relative mortality in the lowest vitamin D category compared to the highest in the meta-analysis of the CHANCES cohorts can be considered to be a strong association when keeping in mind the heterogeneous nature of the outcome and the comprehensive adjustment [21].

### 4.2. Consistency

The association of low 25(OH)D and all-cause mortality was observed with quite high consistency because the individual participant data meta-analysis in the CHANCES consortium observed a significant association with low heterogeneity among study results [21].

### 4.3. Biological Gradient

A dose-response relation of decreasing mortality with increasing 25(OH)D levels was observed in both the CHANCES and the ESTHER analysis [20,21].

### 4.4. Temporality

Temporality is generally guaranteed in prospective cohort studies.

### 4.5. Plausibility

An effect of vitamin D deficiency on a variety of diseases is biologically plausible because the vitamin D receptor is expressed in a large number of tissues and on several immune cells [6,39]. Potential mechanisms linking vitamin D deficiency to diseases, including CVD, cancer, diabetes, respiratory and infectious diseases have been reviewed elsewhere [40,41,42,43,44,45]. However, as outlined in chapter 3, the results from the ESTHER study speak against a causal role of vitamin D in the development of the major chronic diseases CVD and cancer and rather suggest that vitamin D might be a prognostic factor in the course of the diseases. There are plausible explanations for a role of vitamin D in the prognosis of diseases that involve the immune system which are outlined in detail in the following chapter 5. 

### 4.6. Specificity

As mentioned in the introduction, vitamin D deficiency is not related to one specific disease. Moreover, in the ESTHER study, vitamin D deficiency was strongly associated with most of the analysed causes of death (Figure 1) [19]. This finding is supported by another study, which observed such wide-spread, unspecific associations of low 25(OH)D [46]. However, with the widespread distribution of the vitamin D receptor in the human body, all these associations could be biologically plausible.

### 4.7. Coherence

A causal association should not be in conflict with generally known facts about the natural history of a disease [38]. The authors of this review are not aware of such a conflict for the association of vitamin D deficiency with mortality and its underlying diseases. 

### 4.8. Experiment

An eligible experiment for deductions on causality is the randomized controlled trial (RCT), which excludes potential confounding by randomization [47]. A systematic review of RCTs conducted in the framework of the acknowledged Cochrane Collaboration and a more recent review already confirmed that vitamin D supply has an effect on mortality [48,49]. However, the effects were weaker than suggested by observational studies, and vitamin D supplementation seemed to be only effective for the administration of vitamin D_3_ in subjects with low 25(OH)D levels at baseline [48]. Mortality was only a secondary outcome in most RCTs, initially designed to study the effects of vitamin D and calcium supplementation on osteoporotic outcomes. New RCTs with a focus on non-skeletal outcomes are needed. Four large trials addressing different non-skeletal outcomes have started in 2012 and are described elsewhere [50]. Among these trials, the Vitamin D and Longevity (VIDAL) trial will be particularly informative with respect to mortality outcomes because it will be specifically designed to assess these endpoints in older adults (65–84 year-old citizens of Great Britain). The first results of these RCTs are expected between 2017 and 2020.

### 4.9. Analogy

The widespread associations of vitamin D deficiency with diseases and mortality could be analogous with the formerly postulated preventive effects of antioxidant supplements (e.g., vitamin A, C and E) for a long list of diseases and mortality, which finally failed the proof of causality in RCTs [51]. However, this analogy criterion is not one of the important ones because vitamin D could also be analogous with smoking, which was also postulated to be associated with several diseases, including cancer and cardiovascular diseases, and these relationships were confirmed in later studies [52]. 

In summary, there are many arguments for a causal association of vitamin D deficiency and increased mortality, but the current evidence cannot rule out remaining doubts because the evidence is mainly based on observational studies and RCTs with serious limitations. Further discussions of Hill’s criteria for the relationship of vitamin D with CVD and cancer can be found elsewhere and come to similar conclusions [53,54,55]. The main limitation of the observational studies is that associations may be confounded by unconsidered factors. The RCTs reported conflicting results and were not specifically designed to address the study question whether vitamin D supplementation has an effect on mortality. Therefore, results from adequately designed RCTs, like the VIDAL study, have to be awaited before final conclusions can be made.

## 5. The Resilience Factor Hypothesis

The results outlined in this review point into the direction that vitamin D deficiency may not be a risk factor for the major chronic diseases CVD and cancer but may be a risk factor for fatal outcomes of these diseases, *i.e.*, death due to stroke, myocardial infarction and cancer in subjects with a history of cancer. The authors of this review hypothesize that 25(OH)D levels could be a marker of resilience to fatality of potentially fatal diseases or conditions. This hypothesis is independent from the question of causality because such a resilience marker could be causally related to mortality or could just be correlated with other resilience factors. The observed unspecific associations of 25(OH)D levels with most of the analysed causes of death in the analysis of the ESTHER study (Figure 1) can lead to a potential explanation of the “resilience factor hypothesis” [19]. A resilience factor, which prevents from all major causes of death, is an adequate function of the immune system. It is known that the immune system regulates vitamin D metabolism and, in turn, 1,25(OH)_2_D modulates the immune system function [56]. In detail, 1,25(OH)_2_D has been ascribed immunosuppressive effects with reduction of lymphocyte proliferation and inhibition of the production of pro-inflammatory cytokines [45,57]. A recent large study (*n* = 957) in older Irish adults (>60 years) observed significant inverse associations of 25(OH)D levels with the inflammatory markers Interleukin-6 (IL-6) and CRP [58]. Furthermore, vitamin D deficiency was associated with a pro-inflammatory profile (determined by the IL-6 to Interleukin-10 (IL-10) ratio). Not all other studies addressing associations of 25(OH)D levels with IL-6 and CRP have observed these associations, but they were mostly conducted in younger and therefore healthier populations [58]. The authors of the Irish study therefore suggest that the association of 25(OH)D with a pro-inflammatory state may be restricted to individuals with some form of chronic disease. Support for this view comes from a recent review of RCTs that aimed to reduce inflammatory markers by administering vitamin D [59]. Overall, 17 trials found reduced inflammatory markers and 19 did not. However, in study populations with highly inflammatory conditions, 6 out of 7 trials found significantly reduced inflammatory markers after vitamin D intake. If this could be verified in further studies, it would be a strong argument for the “resilience factor hypothesis”: low 25(OH)D levels can be a risk factor for fatal events of chronic diseases because they are negatively correlated with pro-inflammatory markers such as CRP, which have been shown to predict survival in subjects with CVD [60,61] or cancer [62,63]. 

In summary, there are two competing explanations how 25(OH)D levels could be associated with fatal but not with non-fatal diseases: 

(1) The causal explanation: Sufficient levels of 1,25(OH)D are needed as a regulator of the response of the immune system when it is challenged by severe diseases to prevent a fatal course of the disease (e.g., by down-regulation of the production of pro-inflammatory cytokines causing chronic inflammation).

(2) The non-causal explanation: When the immune system is challenged by severe diseases, the production of vitamin D metabolites is down-regulated by the immune system for an unknown reason. The response of the immune system determines the fatality of the disease (e.g., by production of pro-inflammatory cytokines causing chronic inflammation) and low 25(OH)D levels are just an unrelated by-product.

To decide between these possible competing explanations, more basic research is needed to understand the relationship of 25(OH)D and the immune system in serious disease conditions. This research should elucidate whether low 25(OH)D levels in serious disease conditions are an unrelated byproduct of a pro-inflammatory immune system response to the condition or whether sufficient levels of 1,25(OH)D are needed as a regulator for an adequate response of the immune system. Furthermore, the question of causality could best be answered by RCTs assessing the efficacy of vitamin D supplementation on mortality.

## 6. Public Health Implications

If a causal association of 25(OH)D levels and mortality could be established, this could have important public health implications because vitamin D deficiency and insufficiency are very common. In the 50–75 year old population of the ESTHER study, the prevalences were 15% for vitamin D deficiency and 44% for vitamin D insufficiency (taken together, 59% of the population had at least insufficient vitamin D levels) [20]. The authors of a Cochrane Review on the efficacy of vitamin D supplementation estimated that one premature death could be prevented by supplementation of 161 subjects with vitamin D_3_ [48].

As vitamin D insufficiency can mostly not be overcome by dietary intake of food rich in vitamin D during winter [39], making vitamin D supplements readily available to everyone at low costs and to organize the distribution could be a solution [64]. However, besides vitamin D supplementation, there are several other approaches by which serum 25(OH)D levels could be increased at the population level [65]. One approach, which has for long been followed in the USA, is to fortify a variety of commonly consumed foods with vitamin D [66]. A second approach is to encourage well-dosed sun-exposure, with doses that are sufficient for vitamin D production (such as 15 min spent outdoors around noon three times a week during summer months) without increasing risk of skin cancer to any relevant extent [67]. Ideally, this should be combined with some outdoor physical activity, whose beneficial health effects are well established [68]. In some regions, UV-B radiation in winter is too low to induce vitamin D production in the skin, though, and exposure of the skin to light from special lamps producing light rich in the UV-B spectrum could be a solution to replace the lack of UV-B radiation from the sun [69]. The risk of adverse events by overdoses of vitamin D (mainly hypercalcemia) appears to be low as previous studies demonstrated that doses of up to 10,000 IU day^−1^ can be safely administered [70]. However, the US-American Institute of Medicine (IOM) recommends a more cautious upper intake level of 4000 IU day^−1^ for adults [71,72], which is about the 6.5-fold dose of the recommended daily intake of 600 IU day^−1^ [27,72].

All of these approaches might be easily implemented, have low costs, a low risk for adverse events and might have the potential to increase the life expectancy of vitamin D deficient individuals. However, as mentioned before, large-scale trials consistently demonstrating this effect have to be conducted before these interventions can generally be recommended.
